# Prevalence of sickle cell disorders and malaria infection in children aged 1–12 years in the Volta Region, Ghana: a community-based study

**DOI:** 10.1186/s12936-020-03500-5

**Published:** 2020-11-23

**Authors:** Mavis Oppong, Helena Lamptey, Eric Kyei-Baafour, Belinda Aculley, Ebenezer Addo Ofori, Bernard Tornyigah, Margaret Kweku, Michael F. Ofori

**Affiliations:** 1 Department of Epidemiology and Biostatistics, School of Public Health, University of Health and Allied Sciences, Hohoe, Ghana; 2 Department of Immunology, Noguchi Memorial Institute for Medical Research, College of Health Sciences, University of Ghana, Accra, Ghana

**Keywords:** Sickle cell disorders, *Plasmodium falciparum*, Malaria, Children, Volta region, Ghana

## Abstract

**Background:**

Alterations in the structure of haemoglobin (Hb) are usually brought about by point mutations affecting one or, in some cases, two codons encoding amino acids of the globin chains. One in three Ghanaians are said to have sickle cell disorders, whereas malaria continues to be one of the leading causes of mortality among children. This study determined the prevalence of sickle cell disorders and malaria infection among children aged 1–12 years in the Volta Region.

**Methods:**

This was a community-based cross-sectional survey that involved 938 children aged 1–12 years selected from three districts, one each from the 3 geographical zones of the Volta Region using a multistage sampling method. Demographic information was collected using a standard questionnaire and anthropometric indices were measured. Isoelectric focusing (IEF) electrophoresis was used to determine the Hb genotypes and sub-microscopic parasites were determined by PCR.

**Results:**

The prevalence of sickling screening positive was 16.0% with an overall prevalence of sickle cell disorders being 2.0%. Among the individual genotypes making up the sickle cell disorders, genotype HbSF was the highest (0.9% as compared to 0.2%; HbSS, 0.6%; HbSC and 0.3%; HbSCF). Microscopic *Plasmodium falciparum* parasitaemia was detected among 5.5% of the children and 14.2% sub-microscopic prevalence by PCR. Children with sickle cell disorders were more likely to have sub-microscopic parasitaemia (AOR = 5.51 95%CI (2.15, 14.10), *p* < 0.001) as well as anaemia (AOR = 3.03 95% CI (1.04, 8.82), *p* = 0.042), compared to those with normal genotypes. There was no significant difference observed between sickle cell disorders and growth and development of the children screened.

**Conclusions:**

Sickle cell disorders were significantly associated with sub-microscopic parasitaemia as well as anaemia in this study. Establishment of sickle cell clinics in the district and regional hospitals will help in the management of children with the disorder and also generate a national database on sickle cell disorders. National neonatal screening policies must also be put in place to help in early detection and management of these disorders.

## Background

Sickle-cell disorders are types of haemoglobin disorders, which occur due to mutations in one of the globin subunits of haemoglobin (Hb), resulting in a change in amino acid sequence [1]. Normal adult haemoglobin (HbA), consists of a combination of 2 β-globin and 2 α-globin chains with a haem [2]. Examples of haemoglobin genotypes considered as sickle cell disorders are sickle cell anaemia/disease (SCA/SCD) (HbSS), sickle cell trait (HbAS), HbSC disease, sickle β-thalassemia disease (S/β thal), HbS with other Hb variants (like D, O-Arab etc.) and HbS/Hereditary persistence of fetal haemoglobin HbS/HPFH (HbSF) [1, 3].

The sickle haemoglobin (HbS) in sickle cell disorders results from an amino acid substitution at the sixth residue of the β-globin subunit; valine for glutamic acid [1, 4]. The resulting valine is a hydrophobic amino acid unlike the glutamic acid, which is hydrophilic. This hydrophobic amino acid interacts with another haemoglobin leading to the polymerization of deoxygenated red blood cell, causing the shape of the normal haemoglobin to be distorted into sickle shaped, which results in the obstruction or occlusion of the blood vessels [5, 6]. Manifestations of sickle cell disease in children include, but not limited to, anaemia, poor growth, fatigue and jaundice [7]. Some common complications include frequent attacks of severe pain also called crises, acute severe anaemia, malaria and other infections, stroke (especially among children), acute lung damage, development of cerebrovascular disease and cognitive impairment [7, 8].

Sickle cell disease patients in the developed world account for only 10% of the world’s SCD patient population [9]. However, it is known that the disorder follows a more severe clinical course in Africa than in other parts of the world, and that infectious diseases (like malaria) have a role in causing this increased severity [8]. In 2010, an estimated 79% of newborns with SCD occurred in sub-Saharan Africa and this proportion is expected to increase to 88% by 2050 [10].

*Plasmodium falciparum* malaria results in high morbidity and mortality among first time primigravid women and children under 5 in sub-Saharan Africa, hence, still remains a major public health problem. It has been hypothesized that, these sickle cell disorders, have been naturally selected to confer protection against severe forms of malaria in endemic areas [11, 12].

Studies have shown that individuals with HbSS or HbAS variants have reduced risk of high-density parasitaemia. However, HbSS individuals have an increased mortality rate if they become parasitaemic compared to HbAS [9, 13]. Those with HbAS have been shown to have asymptomatic infections and are best described as malaria-protective [8, 14, 15]. The mechanisms proposed involves reduced parasite growth in infected erythrocytes, decreased cytoadherence of parasitized RBCs to endothelium, which enhances the removal of parasitized cells by the immune system [16].

HbSC disease is associated with significant clinical manifestations, but milder than those of HbSS [1, 8]. Individuals with HbSC disease have a higher mean Hb than those with HbSS [17]. Although HbSF has ~ 70% HbS and ~ 30% HbF, it is clinically asymptomatic compared to HbSS because every RBC contains ~ 30% HbF. Since HbF does not participate at all in polymer formation, a situation where hydrophobic amino acid interacts with another haemoglobin to aggregate into large polymers, thus prevents sickling of all RBCs under physiological conditions [1]. HbSS has more severe clinical manifestations, thus it is of great public health concern, compared to HbSC and HbSF that has mild clinical symptoms [10].

In Ghana, studies indicate that 2% of Ghanaian newborns are affected by SCD annually; one in three Ghanaians has HbAS, HbSS or HbSC genotype [18, 19]. Newborn screening with early diagnosis and comprehensive care has been shown to improve child survival since the sickle cell disease has a high mortality rate in the first few years of life [18, 20, 21]. A previous study in one of the Municipalities in the Volta Region of Ghana discovered a considerable prevalence of sickle cell disorders among children [22] and malaria prevalence of 28% in this region. This study determined the prevalence of sickle cell disorders among children aged 1–12 years, and also assessed the influence of these disorders on malaria infection, anaemia and the growth and development of children.

## Methods

### Study sites and population

**Fig. 1:**
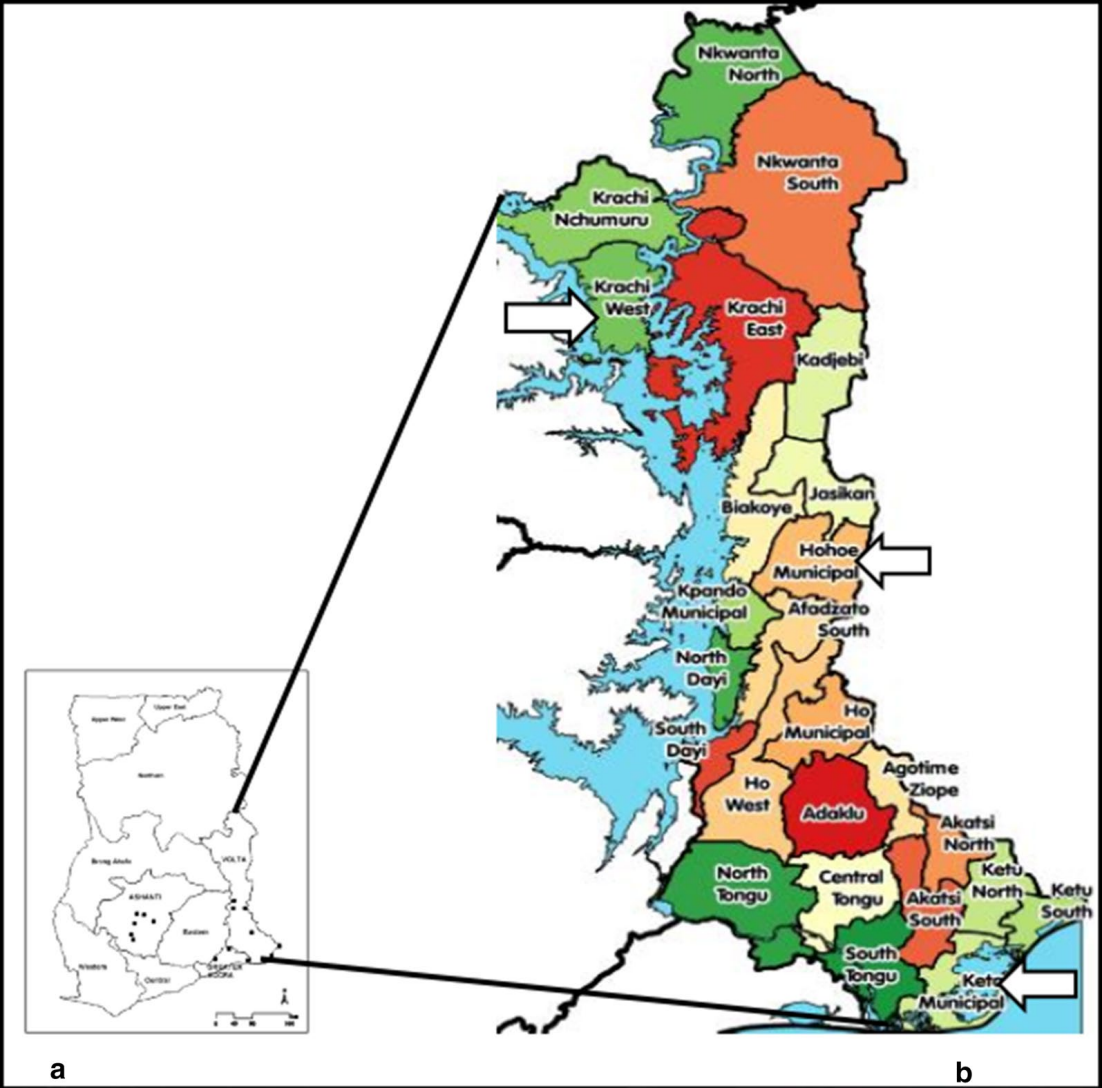
Map of Ghana (**a**) showing the Volta Region with all its districts (**b**). The Study sites (Keta Municipality, Hohoe Municipality and the Krachi West district) are shown by the white arrows

The study was conducted in 3 out of 25 districts of the Volta region (one from each of the 3 ecological zones). The selected districts were Keta Municipality (Southern zone), Hohoe Municipality (Middle zone) and Krachi West District (Northern zone) (Fig. 1).

Keta Municipality has a population of approximately 147,618 with more than half (53.3%) living in urban areas. It has annual rainfall of less than 1000 mm, being one of the driest along the coast of Ghana. The population of Hohoe Municipality is about 167,016 with 52.6% of this populace living in urban areas. The annual rainfall is 1592 mm. Krachi West District has a population of 49,417 with 81.8% of the natives living in rural areas. A map of the region indicating the sites is shown (Fig. 1).

Malaria transmission in the Volta region reflect the national transmission pattern and differs among the three major bioecological zones in Ghana. Keta is in the coastal scrub zone, Hohoe within the middle belt with forest transition or semi-deciduous zone and Krachi representing the northern Guinea savanna zone. Transmission is low in Keta, intermediate in Hohoe and Krachi has high transmission [23].

The study was conducted in December 2018, and the study population consisted of children aged 1–12 years living in selected communities within the 3 selected districts. Inclusion criteria were children within the stated years whose parents/guardians gave consent to be part of the study and were residents in the study area. Those excluded were children who were seriously ill and requiring medical care, and those with major congenital defects as well as those whose parents/guardians did not consent to be part of the study.

### Study design and sampling

Cross-sectional community-based surveys were used. A multi-staged sampling was used in which the list of all the districts in each zone was obtained and one district randomly selected from each zone. Three communities were randomly selected from each of the 3 selected districts giving a total of nine communities. However, during the survey, one community did not confirm their willingness to participate in the study, hence 8 communities out of 9 were visited. Interviews were conducted and recorded using semi-structured questionnaires to collect background demographic information of children. In each of the selected communities, all children who were eligible and their parents/guardians gave consent, were enrolled. Anthropological measurements and biological samples were collected from 938 participants.

### Data collection and sampling

Parents/guardians of the children were interviewed using a pre-tested semi-structured questionnaire. Finger prick dried blood spots (DBS) were collected on Whatman No.5 filter paper (GE Healthcare, England), for Hb genotyping and PCR. The filter paper blots were air-dried and stored with desiccant at 4ºC until ready to be used. Some blood drops were also collected on two frosted slides, one for malaria microscopy (thick and thin films) and the other for sickling screening test.

### Anthropometric and axillary temperature measurement

Height was measured using a non-stretchable tape to the nearest 1 cm according to World Health Organization (WHO) standard. Weight measurement was taken with minimal clothing and without shoes, on a weighing scale with the zero-error corrected before each measurement. Mid Upper Arm Circumference (MUAC) was measured to the nearest 1 cm using a non-stretchable tape. The growth and body composition of the study population was assessed using the Z-scores of the anthropometric indices of height-for-age (HAZ), weight-for-age (WAZ) and body mass index- for-age (BAZ). The WHO Anthroplus Software was used for the conversion to enable comparison of the study participants to a reference population [24]. Since standing height was taken for all children due to lack of the appropriate equipment, those less than 24 months (who were supposed to be measured in recumbent position) had 0.7 cm added to their height by the software, to derive an estimated length. All Z-scores which were outside the WHO flags, thus, WAZ − 6 to + 5; HAZ − 6 to + 6; and BAZ − 5 to + 5 were excluded from the data set. Axillary temperature was measured using a non-contact infrared thermometer (UNIONCARE, Model: IT-122).

### Clinical classification

According to the study protocol, fever was defined as an axillary temperature of ≥ 37.5^◦^C. Stunting was defined as HAZ < -2SD, underweight as WAZ < -2SD, thinness as BAZ < -2SD, overweight as BAZ >  + 1SD and obesity as BAZ >  + 2SD (24). Participants who were stunted, underweight, obese and thin were all classified as malnourished. Operational definition for parasitaemia was microscopy positive and that for clinical malaria was any parasitaemia plus fever. Anaemia was classified using the WHO Vitamin and Mineral Nutrition Information System [25]. The classification of Hb disorders were grouped into normal (HbAA), Sickle cell disorders ((HbSS, HbSC, HbSF and HbSCF), Sickle cell trait (HbAS) and “other Hb disorders” (HbAC, HbAF, HbCC and HbACF) as shown in the supplementary (Additional file 1: Table S1). In this study, sickle cell trait was grouped separately from the other sickle cell disorders to ascertain their individual impact on anaemia and malaria.

### Haemoglobin level determination

A drop of finger prick blood was used to determine the haemoglobin level photometrically using a portable haemoglobin analyser (Hemocue, Sweden). Haemoglobin levels were classified using the WHO Vitamin and Mineral Nutrition Information System [25] with slight modifications; all the three different classifications of Anaemia; “Mild”, “Moderate” and “Severe”, were merged under one group, “Anaemia”.

### Sickling screening test and haemoglobin genotyping

Study participants were screened for sickling status using the sodium metabisulfite method (Sigma Aldrich, USA). Haemoglobin genotyping was performed using Isoelectric focusing (IEF) electrophoresis method with the Multiphor II electrophoresis unit (GE Healthcare, Little Chalfont, England). Controls and samples were run at a constant power of 26 W (1200 V and 300 mA) for about 1 h, 30 min. The gel plates were fixed with 10% trichloroacetic acid and then placed in a distilled water washing tray on a racking shaker for 12 min. After 5 cycles of washing, the gel was placed in a 60 oC oven to dry. The bands formed by individual haemoglobin were then compared to those of the respective controls and recorded.

### Parasitaemia by microscopy

Both thick and thin blood smears were prepared on a single frosted slide, air dried and stained with 10% Giemsa (thin film fixed with methanol) for detection of both asexual and sexual stage parasites by microscopy under oil immersion (100× magnification). Parasite densities were assessed by counting 200 leucocytes and were later converted to number of parasites per microlitre of blood by assuming a standard leucocyte count of 8000/μl [26]. A negative slide was one on which no parasites were seen after examination of at least 200 fields.

### Extraction of parasite DNA

Parasite DNA was extracted from the punched DBS using Chelex®-saponin protocol as described elsewhere [27–29] with little modifications. In summary, 50 µl of 10% Saponin and 1 ml Phosphate Buffered Saline (PBS at pH 7.2) were added to each punched DBS in an Eppendorf tube and left overnight at 4 °C. The filter paper was washed twice in 1 mL PBS and then heated up to 100 oC in 150 µl of sterile distilled water and 50 µl of 20% Chelex®-100 (Sigma-Aldrich, USA) for 10 min, with intermittent vortexing. After the final centrifugation step (13,000 rpm for 10 min), the extracted DNA was transferred into a 96-well PCR plate, covered with a plate sealer and stored at -20 °C for PCR analysis.

### Molecular detection of *Plasmodium falciparum* parasites

*Plasmodium falciparum* was detected by a nested PCR amplification, as described previously [30] with minimal modification. The primary reaction mixture was 15 µL and contained 2μL of DNA, 0.20 µM of each rPLU5 and rPLU6 primers (Eurofins Genomics, Germany), 200 µM of each of the four deoxyribonucleotide triphosphates (dNTPs) (Promega Corporation, USA), 3 mM MgCl_2_ (Sigma-Aldrich, USA) and 0.05 units/µL of *Thermus aquaticus* (Taq) DNA polymerase (Sigma-Aldrich, USA). The reaction cycling parameters comprised of an initial denaturation at 94 °C for 2 min, denaturation at 94 °C for 1 min, annealing at 55 °C for 1 min 30 s (all for 30 cycles). Extension was at 72 °C for 3 min, and a final extension at 72 °C for 5 min. The secondary reaction mixture was similar to the primary. However, 1 μL of the primary PCR product was used as DNA template and with rFal1 and rFal2 primer set used in the amplification. For nest 2 reaction, the cycling conditions used were as follows, initial denaturation at 95 °C for 1 min, was followed by denaturation at 94 °C for 1 min, annealing at 55 °C for 1 min 30 s (all for 30 cycles). Extension was at 72 °C for 2 min, and a final extension at 72 °C for 5 min. The PCR products were stored at 4 °C until analysis. Positive controls and no DNA template negative control were part of each set of reaction.

### Molecular detection of *Plasmodium malariae* parasites

Detection of *Plasmodium malariae* was done according to protocol by Padley and colleagues [31] with some slight modifications. A single reverse primer (Prv) with conserved regions for all four *Plasmodium* species was used with the species-specific forward primer for *P. malariae* (Pm-F). The reaction mixture contained 0.20 µM of each oligonucleotide primers, 200 µM of each dNTPs, 3 mM MgCl_2_ and 0.05 units/µL of Taq DNA polymerase. Two microlites of DNA was used as the template in a total reaction volume of 15 µL. Cycling conditions were carried out as follows, initial denaturation at 95 °C for 5 min, denaturation at 95 °C for 30 s, annealing at 56.2 °C for 30 s (35 cycles). Extension was at 72 °C for 1 min, and a final extension at 72^o^ C for 5 min. The PCR products were stored at 4 °C until analysis. Positive controls and no DNA template negative control were part of each set of reaction.

### PCR product analysis

PCR products were resolved using a 2% agarose gel stained with 0.5 μg/mL ethidium bromide electrophoresis. The digital images were then analysed with a Gel Dock (VILBER, France). Gel photographs were scored by visual comparison of DNA fragments for individual samples and estimated using 100 base pair (bp) DNA ladder marker (Promega Corporation, USA). A sample was considered positive if a 205 bp product was detected for *P. falciparum* and 412 bp detected for *P. malariae.*

### Statistical analysis

Data collected was double-checked before entering onto Epi Data version 3.1 (Odense, Denmark). They were then exported to Stata version 15.0 (StataCorp LLC, Texas, USA) for analysis. Frequency of various findings were expressed as percentages. Descriptive data were summarized as means or proportions. Demographic characteristics (age group, gender, ethnicity, and district), malariometric and anthropometric parameters as well as anaemia were compared among the Hb classifications using Chi- square or Fischer’s exact tests (when the frequencies were less than 5). Binary as well as multi-variate logistic regressions were performed to test for the strength of association observed. Statistical significance was defined as p < 0.05.

## Results

### Demographic and clinical characteristics of children

A total of 938 samples from the three districts were analysed. Hohoe Municipality had the highest number of participants 337 (35.9%), 289 (30.8%) were from Keta, and 312 (33.3%) from Krachi West. The mean age of the children was 6.4 ± 3.4 years, with 52.6% being females. Majority of the Children 579 (61.7%) were aged 5 to11years as compared to the other age groups. Among the ethnic groups, the Ewes constituted 46.8% (439). Majority of the children 850 (90.6%) slept inside LLINs the night before the survey as compared to less than 10% who did not use bed nets the night before the survey (p < 0.001) (Table 1).

**Table 1:** Socio-demographic characteristics of study participants stratified by district

Variable	Hohoe (n = 337)	Keta (n = 289)	Krachi (n = 312)	Total (n = 938)	p-value
Gender (%)					
Female	183 (54.3)	147 (50.9)	163 (52.2)	493 (52.6)	
Male	154 (45.7)	142 (49.1)	149 (47.8)	445 (47.4)	0.6855
Mean age (sd)	5.6 (3.3)	7.0 (3.0)	6.7 (3.6)		** < 0.0004**
Age categories (%)					
1–4yrs	136 (40.3)	66 (22.8)	100 (32.1)	302 (32.2)	
5–11yrs	185 (54.9)	203 (70.2)	191 (61.2)	579 (61.7)	
Above 12 years	16 ((4.7)	20 (7.0)	21 (6.7)	57 (6.1)	**0.0002**
Ethnicity (%)					
Akan	8 (2.4)	7 (2.4)	10 (3.2)	25 (2.7)	
Ewe	133 (39.5)	281 (97.2)	25 (8.0)	439 (46.8)	
Guan	33 (9.8)	0 (0.0)	148 (47.4)	181 (19.3)	
Others	163 (48.3)	1 (0.4)	129 (41.4)	293 (31.2)	** < 0.0004**
Bed net use (%)					
No	48 (14.2)	15 (5.2)	25 (8.0)	88 (9.4)	
Yes	289 (85.8)	274 (94.8)	287 (92.0)	850 (90.6)	**0.0003**
Mean Parasite Density/µ L(sd)	20,983.5 (64,810.2)	7255.7 (12,667.2)	100,808.6 (191,698.9)		**0.0406**
Mean Hb g/dL(sd)	11.2 (1.7)	10.5 (1.3)	11.6 (1.6)	11.1 (1.6)	** < 0.0004**
HAZ					
Normal	254 (75.4)	234 (81.0)	259 (83.0)	747 (79.6)	
Stunted	83 (24.6)	55 (19.0)	53 (17.0)	191 (20.4)	**0.0430**

### Phenotypic and genotypic prevalence of sickle cell disorders

#### Sickling status by Sodium Metabisulfite screening test

Out of the participants screened, 16.0% (150/938) were positive by sodium metabisulfite sickling screening test. Among the female participants, 15.4% (76/493) of them were sickling positive with 16.2% (49/302) of the children under 5 years also being sickling positive phenotypically. There was no significant difference in the number of sickling positives by metabisulfite screening test from the three districts (*p* = 0.2406). There was no significant difference between the districts, as well as age groups, gender and bed net usage when stratified by sodium metabisulfite test (Table 2).

**Table 2:** Demographic characteristics of the study participants stratified by metabisulfite screening test

Variable	Negative (n = 788)	Positive (n = 150)	Total (n = 938)	*p*-value
District (%)				
Hohoe	279 (35.4)	58 (38.7)	337 (35.9)	
Keta	238 (30.2)	51 (34.0)	289 (30.8)	
Krachi	271 (34.4)	41 (27.3)	312 (33.3)	0.2406
Gender (%)				
Female	417 (52.9)	76 (50.7)	493 (52.6)	
Male	371 (47.1)	74 (49.3)	445 (47.4)	0.6766
Age (%)				
1–4yrs	253 (32.1)	49 (32.7)	302 (32.2)	
5–11yrs	486 (61.7)	93 (62.0)	579 (61.7)	
Above 12 yrs	49 (6.2)	8 (5.3)	57 (6.1)	0.9154
Bed net use (%)				
No	77 (9.8)	11 (7.3)	88 (9.4)	
Yes	711 (90.2)	139 (92.7)	850 (90.6)	0.4319

#### Sickling status by genotypic analysis

**Fig. 2:**
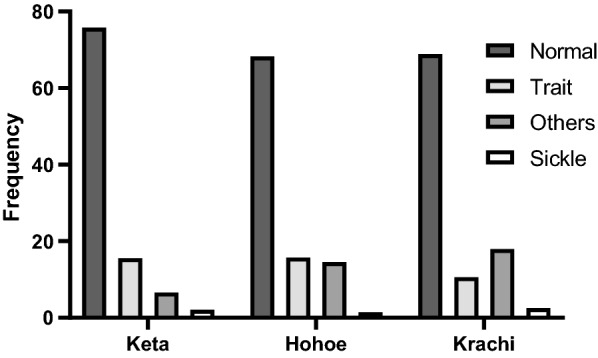
Prevalence of haemoglobin classifications within the study population. Normal = Hb AA, Trait = HbAS, Sickle Cell Disorders = HbSS, HbSC, HbSF and HbSCF and Other Hb Disorders = (HbAC, HbCC, HbAF and HbACF). The error bars represent 95% CI for prevalence of Hb classification (*p* < 0.001)

Using electrophoresis, all the samples were successfully genotyped and the Hb genotypes detected in this study were predominantly the HbAA type (70.8%; 664/938), followed by the heterozygous HbAS (14.0%; 131/938) and HbAC (10.8%; 101/938). The minor genotypes detected were HbAF (1.9%; 18/938), HbCC (0.4%; 4/938), HbSC (0.6%; 6/938), HbSF (0.9%; 8/938) and HbSS (0.2%; 2/938). In addition to these genotypes, HbACF (0.1%; 1/938) and HbSCF (0.3%; 3/938) genotypes that had three separate bands were detected. The overall prevalence of sickle cell disorders (HbSS, HbSC, HbSF and HbSCF) in this study was 2.0%. The difference in the distribution of normal genotype, sickle cell disorders, sickle cell trait and “other Hb disorders” among the 3 districts was statistically significant (*p* < 0.001) (Fig. 2). Sodium Metabisulphite test is used to differentiate cells which have sickle haemoglobin from those that do not have. However, the IEF is used to determine the exact genotypes of these cells, hence the difference in the numbers.

### Malariometric indices

The overall prevalence of *P. falciparum* infection by microscopy in the study population was 5.5% (52/938), whereas sub-microscopic *P. falciparum* infection by PCR was 14.2% (133/938). Fever was present in 10.1% (95/938) of the participants. Children with sickle cell disorders had a higher proportion of sub-microscopic parasitaemia compared to those with other conditions (*p* = 0.0004) (Table 3). Factors predicting sub-microscopic carriage in the three districts were analysed using multiple logistic regression with sub-microscopic parasitaemia as a binary outcome. It was observed that children with sickle cell disorders were 5.51 times more likely to have sub-microscopic parasitaemia, compared to those with normal genotype (AOR = 5.51: 95% CI (2.15,14.10), *p* < 0.001). Also, children in the 5-11 year group are more likely to have sub-microscopic parasitaemia compared to children under five (5) years (AOR = 1.87:95%CI (1.20,2.90), *p* = 0.006). The geographical location (district), and gender did not influence carriage of sub-microscopic parasitaemia (Table 4).

**Table 3:** Haemoglobin classification and malaria indices

Variables	Normal (n = 664)	Other Hb disorders (n = 124)	Sickle cell disorders (n = 19)	Sickle cell trait (n = 131)	Total (n = 938)	*p*-value
Sub-microscopic parasitaemia (%)						
Negative	573 (86.3)	110 (88.7)	10 (52.6)	112 (85.4)	805 (85.8)	
Positive	91 (13.7)	14 (11.3)	9 (47.4)	19 (14.5)	133 (14.2)	**0.0004**
Parasite by microscopy (%)						
Negative	626 (94.3)	121 (97.6)	17 (89.5)	122 (93.1)	886 (94.5)	
Positive	38 (5.7)	3 (2.4)	2 (10.5)	9(6.9)	52 (5.5)	0.2965
Fever (%)						
Fever	76 (11.4)	10 (8.1)	3 (15.8)	6 (4.6)	95 (10.1)	
No fever	588 (88.6)	114 (91.9)	16 (84.2)	125 (95.4)	843 (89.9)	0.0736
Mean haemoglobin (SD) (g/dL)	11.2 (1.6)	11.4 (1.7)	9.1 (2.4)	10.9 (1.5)	11.1 (1.6)	** < 0.0004**

**Table 4:** Factors influencing sub-microscopic parasitaemia among study participants

Variables	Sub-microscopic parasitaemia		*χ*^2^ (*p* value)	COR (95% CI), *p* value	AOR (95% CI), *p* value
Sex	Negative	Positive			
Female	417 (51.8)	76 (57.1)		Ref.	
Male	388 (48.2)	58 (42.9)	1.11 (0.291)	0.82 (0.57, 1.18), 0.292	
Age group					
1–4	274 (34.0)	28 (21.5)		Ref.	Ref.
5–11	481 (59.8)	98 (73.7)		1.92 (1.24, 2.99), 0.004	1.87 (1.20, 2.90), **0.006**
≥ 12	50 (6.2)	7 (5.3)	8.95 (0.011)	1.29 (0.54, 3.11), 0.567	1.14 (0.46, 2.80), 0.777
District					
Keta Municipality	253 (31.4)	36 (27.1)		Ref.	
Hohoe Municipality	291 (36.2)	46 (34.6)		1.11 (0.70, 1.77), 0.659	
Krachi West	261 (32.4)	51 (38.4)	2.288 (0.319)	1.40 (0.88, 2.21), 0.150	
Hb classification					
Normal	573 (71.2)	91 (68.4)		Ref.	Ref.
Trait	112 (13.9)	19 (14.3)		1.06 (0.63, 1.82), 0.809	1.07 (0.63, 1.84), 0.793
Other Hb disorders	110 (13.7)	14 (10.5)		0.88 (0.48, 1.82), 0.608	0.86 (0.47, 1.54), 0.605
Sickle cell disorders	10 (1.2)	9 (6.8)	17.72 (0.001)	5.67 (2.24, 14.32), < 0.001	5.51 (2.15, 14.10), < **0.001**

## The association of anaemia and sickle cell disorders among children

**Fig. 3:**
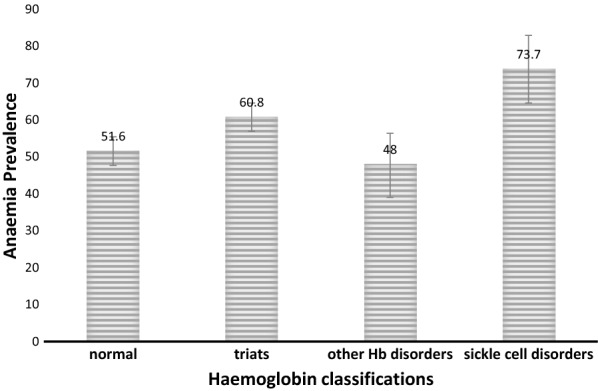
Prevalence of anaemia among the children with and without sickle cell disorders. The **e**rror bars show 95% CI of anaemia prevalence among the Hb classifications (*p* = 0.042)

The overall mean Hb measured was 11.1 ± 1.4 g/dL. Children from Keta District had significantly lower Hb (10.5 ± 1.3) compared to Hohoe Municipality (11.2 ± 1.7), and Krachi West District (11.6 ± 1.6) (*p* < 0.0004) (Table 1). The mean Hb for those with sickle cell disorders 9.1 ± 2.4 g/dL, was significantly lower than normal (Hb:11.2 ± 1.6 g/dL, sickle cell trait:10.9 ± 1.5 g/dL and other Hb disorders: 11.4 ± 1.7 g/dL). The prevalence of anaemia among children with sickle cell disorders was 73.7% (14/19) (Fig. 3). Sickle cell disorders influenced the anaemia status of the study participants (Table 5).

**Table 5:** Factors influencing anaemia among study participants

Variables	Anaemia		*χ*^2^ (*p* value)	COR (95% CI), p value	AOR (95% CI), *p* value
Sex	No anaemia	Anaemia			
Female	250 (56.3)	243 (49.2)		Ref.	Ref.
Male	194 (43.7)	251 (50.8)	4.64 (0.031)	1.32 (1.03, 1.72), 0.031	1.30 (0.96, 1.67), 0.091
Age group					
1–4	121 (27.2)	181 (36.6)		Ref.	Ref.
5–11	296 (66.7)	283 (57.3)		0.63 (0.48, 0.84), 0.002	0.53 (0.39, 0.72), **<0.001**
≥12	27 (6.1)	30 (6.1)	10.12 (0.006)	0.70 (0.40, 1.23), 0.224	0.59 (0.32, 1.08), 0.086
District					
Keta Municipality	79 (17.8)	210 (42.5)		Ref.	Ref.
Hohoe municipality	176 (39.6)	161 (32.6)		0.34 (0.24, 0.48), <0.001	0.30 (0.21, 0.42), **<0.001**
Krachi West	189 (42.6)	123 (24.9)	71.90 (<0.001)	0.24 (0.17, 0.34), <0.001	0.23 (0.16, 0.33), **<0.001**
Malaria microscopy					
Negative	424 (95.5)	462 (93.5)		Ref.	
Positive	20 (4.5)	32 (6.5)	1.67 (0.196)	1.46 (0.82, 2.59), 0.198	
Sub-microscopic malaria					
Negative	389 (87.6)	416 (84.2)		Ref.	
Positive	55 (12.4)	78 (15.8)	1.81 (0.178)	1.29 (0.89, 1.87), 0.179	
HAZ					
Normal	373 (83.8)	375 (75.9)		Ref.	Ref.
Stunting	72 (16.2)	119 (24.1)	7.15 (0.008)	1.56 (1.12, 2.16), 0.008	1.44 (1.02, 2.05), **0.037**
Hb classification					
Normal	322 (72.5)	342 (69.2)		Ref.	
Trait	52 (11.7)	79 (16.0)		1.45 (1.00, 2.13), 0.056	1.03 (0.68, 1.56), 0.898
Other Hb disorders	65 (14.6)	59 (11.9)		0.86 (0.59, 1.27), 0.461	1.37 (0.91, 2.06), 0.136
Sickle cell disorders	5 (1.1)	14 (2.8)	8.18 (0.042)	2.62 (0.94, 7.38), 0.067	3.03 (1.04, 8.82), **0.042**

### Influence of sickle cell disorders on growth and development of children

From the study, although about 19% of the participants were underweight, and 20% were stunted, no association was observed between the various haemoglobin disorders and the anthropometric indices of the children in this study (Additional file 1: Table S2).

## Discussion

This cross-sectional study sought to determine the prevalence of sickle cell disorders and malaria infection among children aged 1–12 years in the Volta region of Ghana. The ethnical background of the participants showed the Ewes dominating, but that was not surprising because they are the majority within the region [32]. There was a higher number of LLIN use among participants (90.6%) compared to 47% from the Ghana Demographic Health Survey Report from the same community [33], as well as 41.7 and 72.8% in other parts of the region [33–35]. Also, in Asutsuare in the Greater Accra Region, bed net usage was lower (56%) as compared to what was observed in this study. This suggests that education on LLIN usage as part of the malaria control efforts is gradually being accepted in the study communities.

The prevalence of sickling positive was 16.0%, which was higher than 6.0% detected previously among children in Asutsuare, in the Greater Accra Region, the higher prevalence of sickling positive observed in this population, could be due to the higher sickling gene pool found in the Volta Region compared to others [36]. However, among the study population, the number of males found to be sickling positive were higher than females. This was contrary to the findings of Antwi-Baffour and colleagues, that observed a higher prevalence of sickling positives (41.7%), among females compared to males [37]. These differences in prevalence could be due to variations in sample size and the geographical locations of where these studies were done.

The overall prevalence of sickle cell disorders which consists of HbSS, HbSC, HbSF and HbSCF was 2.0%, this was found to be higher than the 0.3–1.2% prevalence levels observed in other studies done in the country [38–40]. The high prevalence observed in this study could be due to the high throughput method used to detect the haemoglobin genotypes. It could also probably be that the sickling gene pool in this study site is higher than those from other sites. The prevalence of the individual genotypes that were classified as sickle cell disorders revealed that genotype HbSC had a higher prevalence than HbSS. This observation is similar to other studies conducted in Ghana [38, 39, 41] and Burkina Faso [42]. It was however contrary to the findings of studies done in Nigeria [43, 44] and in Ghana (Accra) [19, 45]. Interestingly, genotype HbSF was observed in 0.9% of the participants, this genotype is rarely detected in most studies with children, since it has been shown to wane off in early childhood by 6 months of age [46, 47]. A study that reviewed records of children in a referral hospital in Ghana observed levels (0.8%) of HbSF [45] that was very close to what was observed in this study. Though fetal haemoglobin (HbF) levels have been associated with protection of children from severe sickle cell crisis [47, 48], disease severity may increase when it persists into adulthood, thus have implications for sickle cell management.

The prevalence of malaria infection using PCR was higher than that of microscopy (14.0% versus 5.5%). The molecular detection of parasites by PCR has been shown to be more sensitive than microscopy in several studies [36, 38, 49, 50]. Children with sickle cell disorders (HbSS) had a significantly higher proportion of sub-microscopic parasitaemia than those with sickle cell trait (HbAS) and other Hb disorders (*p* = 0.001). This is probably due to immune regulation, that results in the suppression of parasites below microscopic detection limit in sickle cell individuals [38], hence their low parasitaemia. Thus it buttresses the established fact that sickle cell trait confers protection against clinical malaria [1, 12–14]. Also, children with sickle cell disorders were found to be 5.5 times more likely to have sub-microscopic parasitaemia than those with normal genotype (AOR = 5.51 95%CI (2.15, 14.10), *p* < 0.001). A similar observation was made in a study conducted in Ghana, which found HbSC (a sickle cell disorder) to have a higher proportion of sub-microscopic *P. falciparum* infection than those with HbAA (AOR = 4.34 95% CI (1.36–13.9)) [38].

The mean Hb levels recorded was lower in children with sickle cell disorders compared to those with normal genotype (9.1 ± 2.4 against 11.4 ± 1.6 g/dL). In addition, children with sickle cell disorders were 3 times more likely to be anaemic compared to those with normal genotype (AOR = 3.03 95%CI (1.04, 8.82), *p* = 0.042). This is not surprising, because there is evidence that the half-life of haemoglobin in sickle cell individuals is shorter than normal haemoglobin and are rapidly destroyed by the spleen because of their abnormal shape, thus such patients tend to have chronic anaemia [12, 15]. This observation was similar to findings from a study from parts of Ghana [38] but different from what was observed in Burkina Faso, where children with normal genotype had a lower mean Hb than those with HbSS or HbSC [42]. This could be as a result of other infections such as clinical malaria or severe malaria anaemia and malnutrition in these children.

The majority of children screened in this study were stunted. This finding could be an indication of the low socio-economic conditions in the communities surveyed. This observation was similar to what was found in a study conducted in Northern Ghana [51]. In this study, there was no significant association between sickle cell disorders and the growth and development of the children. However, a study conducted in the Ashanti region of Ghana, observed a significantly lower risk of stunting in children with sickle cell trait than among those with HbAA (*p* = 0.035) [39]. Another study conducted in Kinshasa observed a significantly higher proportion of subjects with stunting and underweight among SCA under 12 years (*p* < 0.001) compared to those with HbAA [52].

The limitations of this study were that information on bed net usage was obtained from parents/guardians of the children without direct observation to confirm the availability and use. Also, the study was conducted at one time in the year, the study was not conducted at different malaria transmission seasons in these communities, therefore, the impact of seasonality on malaria and anaemia could not be determined.

## Conclusions

Sickle cell disorders were significantly associated with sub-microscopic parasitaemia infection as well as anaemia. Association between sickle cell disorders and growth and development of study participants could not be ascertained. More sensitive molecular techniques must be used when conducting research on haemoglobin disorders, so as to detect silent genotypes that may be prevalent. Establishment of sickle cell clinics in the district and regional hospitals will help in the management of children with sickle cell disorders as well as to help in generating national data on sickle cell disorders. Also, neonatal screening in all regions of the country will be an effective way for early detection to inform efficient management of these disorders.

## Supplementary information

**Additional file 1: Table S1.** Haemoglobin classification from isoelectric focussing analysis. **Table S2.** Distribution of anthropometric indices among the haemoglobin classifications.

## Data Availability

The datasets analysed for this study are included in the article, additional data required are available from the corresponding author on reasonable request.

## Abbreviations

AA: Normal Haemoglobin
AC: Heterozygous for haemoglobin C
AS: Sickle trait
BAZ: Body mass index- for-age
CC: Homozygote for haemoglobin C
DBS: Dried blood spots
DNA: Deoxyribonucleic Acid
Hb: Haemoglobin
HAZ: Height-for-age
IEF: Isoelectric focusing
SC: Haemoglobins S and C
SS: Sickle cell
Sickle cell disorders: Haemoglobin HbSF, HbSCF, HbACF sickle β-thalassemia disease (S/β thal)
HbS/HPFH: HbS/Hereditary persistence of fetal haemoglobin
HbSF: Fetal haemoglobin
LLIN: Long-Lasting Insecticide Net
MUAC: Mid Upper Arm Circumference
PCR: Polymerase Chain Reaction
REC: Research Ethics Committee
WAZ: Weight-for-age
