# Supplementary material for: Prevalence of sickle cell disorders and malaria infection in children aged 1–12 years in the Volta Region, Ghana: a community-based study
Source: Malar J. 2020 Nov 23;19:426. doi: 10.1186/s12936-020-03500-5 (PMC7684914; doi:10.1186/s12936-020-03500-5)
Supplement: Supplementary file 1 — Additional file 1: Table S1. Haemoglobin classification from isoelectric focussing analysis. Table S2. Distribution of anthropometric indices among the haemoglobin classifications. [file 12936_2020_3500_MOESM1_ESM.docx]

APPENDIX/SUPPLEMENTARY MATERIAL

***Supplementary Table S1: Haemoglobin classification from isoelectric focussing analysis***

| **Classification** | **Genotypes** |
| --- | --- |
| Normal genotype | HbAA |
| Sickle cell trait | HbAS |
| Sickle cell disorders | HbSC, HbSS, HbSF, HbSCF |
| Other haemoglobin disorders | HbAF, HbCC, HbAC, HbACF |

***Supplementary Table S2: Distribution of anthropometric indices among the haemoglobin classifications***

| **Variable** | **Haemoglobin classification** | | | | **Total** | **P value** |
| --- | --- | --- | --- | --- | --- | --- |
|  | **Normal** | **Sickle cell trait** | **Sickle cell disorder** | **Other Hb disorders** |  |  |
| Underweight (WHZ < -2SD) | 88 (17.3) | 26 (25.5) | 3 (21.4) | 25 (25.8) | 142 (19.7) | 0.083 |
| Stunting (HAZ< -2SD) | 129 (19.7) | 30 (23.4) | 5 (26.3) | 27 (22.7) | 191 (20.7) | 0.645 |
| Obesity (BAZ> +2SD) | 20 (3.1) | 2 (1.6) | 1 (5.3) | 1 (0.8) | 24 (2.6) | 0.699 |
| Thinness (BAZ< -2SD) | 95 (14.5) | 17 (13.4) | 3 (15.8) | 16 (13.5) | 131 (14.2) | 0.699 |
